# Compound heterozygous B3GALNT2 mutations in a fetus with encephalocele: A case report

**DOI:** 10.1002/ccr3.8691

**Published:** 2024-04-05

**Authors:** Dandan Ling, Wanqin Xie, Xiao Mao, Shengzhi Yang, Haiyan Pang, Ping Yang, Ping Shen, Yabing Tang

**Affiliations:** ^1^ Clinical Research Center For Placental Medicine In Hunan Province Changsha City China; ^2^ Department of Obstetrics Hunan Provincial Maternal and Child Health Care Hospital Changsha City China; ^3^ NHC key labratory of birth defects for research and prevention, Hunan Provincial Maternal and Child Health Care Hospital Changsha City China; ^4^ Department of Pediatrics Hunan Provincial Maternal and Child Health Care Hospital Changsha City China; ^5^ Department of Reproductive Medicine Affiliated Hospital of Weifang Medical University Weifang China

**Keywords:** B3GALNT2, dystroglycanopathy, Encephalocele, neural tube defects, prenatal diagnosis

## Abstract

An encephalocele is a congenital malformation characterized by protrusion of the intracranial contents through a cranial defect. We report that a fetus of a pregnant mother who had two consecutive pregnancies with ultrasound‐detected encephalocele carried compound heterozygous variants in *B3GALNT2* NM_152490.5:c.[1423C > T (p.Gln475Ter)]; [261‐2A > G] of maternal and paternal origins, respectively, as confirmed by exome sequencing followed by Sanger sequencing validation. The present case implies that mutations in *B3GALNT2*, a well‐known dystroglycanopathy causative gene, may result in a phenotype of neural tube defect, providing new insights into the clinical spectrum of *B3GALNT2*‐related disorders. Our study may contribute to prenatal screening/diagnosis and genetic counseling of congenital brain malformations.

## 1 INTRODUCTION

An encephalocele, is a congenital malformation (neural tube defect) characterized by protrusion of the intracranial contents through a cranial defect. The prevalence of encephalocele ranges from 0.08 to 0.5 per 1000 live births.^1^, ^2^, ^3^, ^4^ Although the causes are largely unknown, a number of pathogenic factors have been reported to be associated with encephalocele, including maternal nutrition deficiencies, inheritance, aflatoxin exposure, advanced paternal age, and long intervals between pregnancies.^5^

The *B3GALNT2* (β‐1, 3‐Nacetylgalactosaminyltransferase 2) gene (OMIM 610194), located on chromosome1q42.3, encodes an enzyme that transfers N‐acetyl galactosamine (GalNAc) in a β‐1,3 linkage to N‐acetylglucosamine (GlcNAc), to form an unique carbohydrate structure (GalNAc‐β‐1–3GlcNAc).^6^ This structure is essential for post‐translational O‐glycosylation of α‐dystroglycan (α‐DG), a heavily glycosylated protein that is widely expressed in a variety of tissues and plays an important role in skeletal muscle and brain development.^7^ Mutations in *B3GALNT2* can result in hypoglycosylation of α‐DG, and have been linked with α‐dystroglycanopathy (α‐DGP), a subtype of congenital muscular dystrophies (CMDs) with autosomal recessive inheritance.^7^, ^8^ Most patients with *B3GALNT2*‐related α‐DGP presented with the most severe WalkerWarburg syndrome (WWS) characterized by a short lifespan or less severe muscle‐eye‐brain disease (MEB).^7^ However, there is a paucity of literature regarding the phenotypes of fetuses carrying *B3GALNT2* variants.

In the present case study, compound heterozygous B3GALNT2 variants were detected in a fetus of a pregnant mother who had two consecutive pregnancies with an encephalocele on ultrasound images. The genotype–phenotype correlation and the implications of the present case for future clinical practice were discussed.

## 2 CASE HISTORY

**FIGURE 1:**
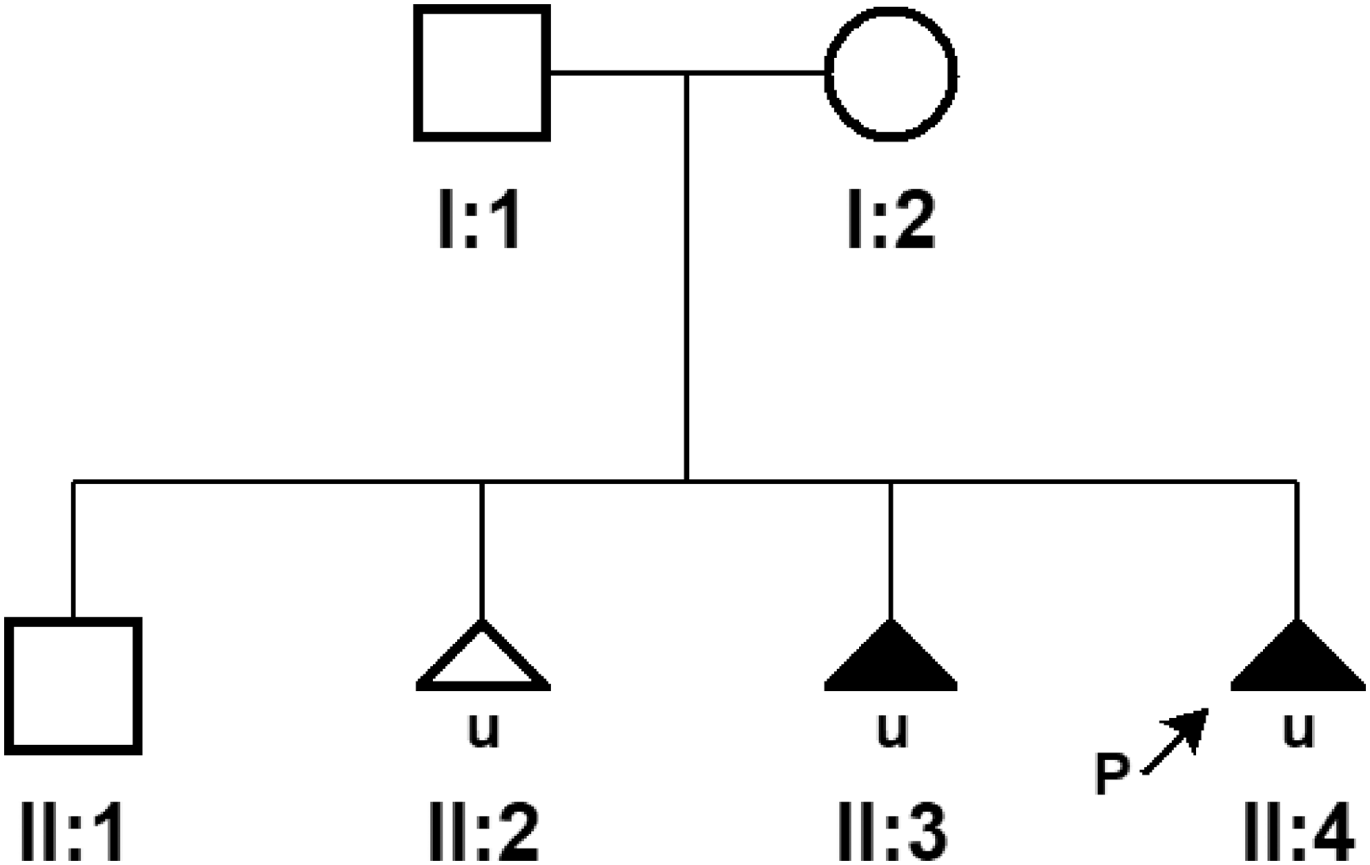
The pregnancy history of the couples in the present study. The proband is indicated with an arrown.

**FIGURE 2:**
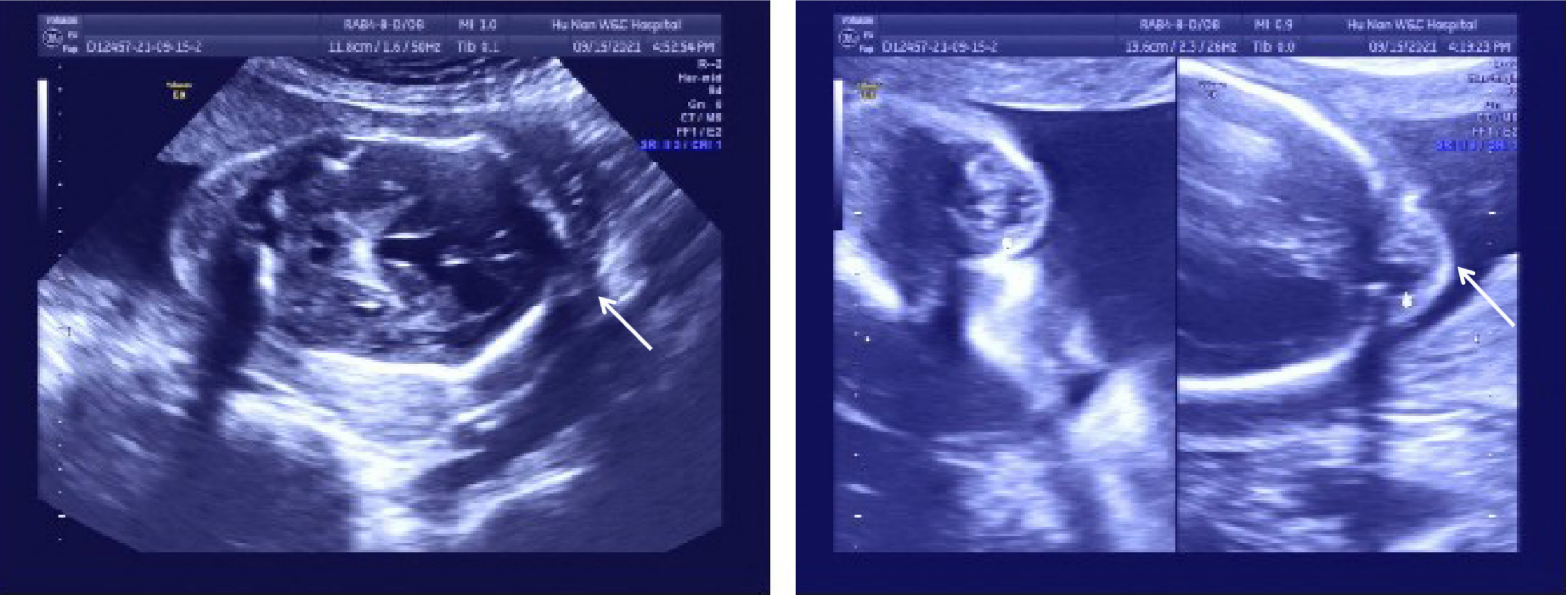
Fetal ultrasound images show the brain structural abnormalities of the third pregnancy (II:3) of the pregnant mother. Fetal skull defect with an encephalocele is indicated with an arrow. NT thickening (8.7 mm), dialation of the third ventricle (6 mm), brain parenchymal echo and cerebellar echo enhancements were also noted.

**FIGURE 3:**
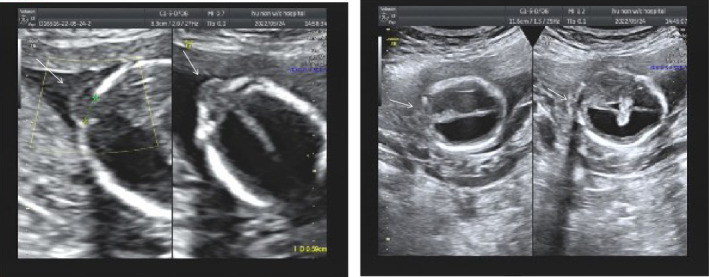
Fetal ultrasound images show the skull defect with an encephalocele (indicated by arrows) at the 17th gestational week in the fourth pregnancy (II:4) of the pregnant mother. NT thickening (6.9 mm) and the enlarged third ventricle were noted.

A 35‐year‐old woman at her 17th weeks in pregnancy presented to the obstetrics department of our hospital. Her medical history showed three previous pregnancies (Figure 1), including the first healthy pregnancy (II:1), missed abortion in the second pregnancy (II:2), and induced labor in the third pregnancy (II:3) due to an encephalocele found by fetal ultrasound examination at the 23rd gestational week (Figure 2). The karyotypes of the pregnant woman and her husband were normal. Her husband and her son's medical histories were not remarkable. Prenatal examinations were prescribed for the currently existing pregnancy (II:4). Fetal ultrasound screening revealed a defect in the skull with encephalocele, bilateral lateral ventricles dilatation (suspected hydrocephalus), and NT thickening (Figure 3).The fourth pregnancy was terminated in the 17th week of gestation by induced labor. With written informed consent, aborted fetal skin tissue and the blood samples of the couple and the son (II:1) were collected for genetic testing.

## 3 METHODS

**FIGURE 4:**
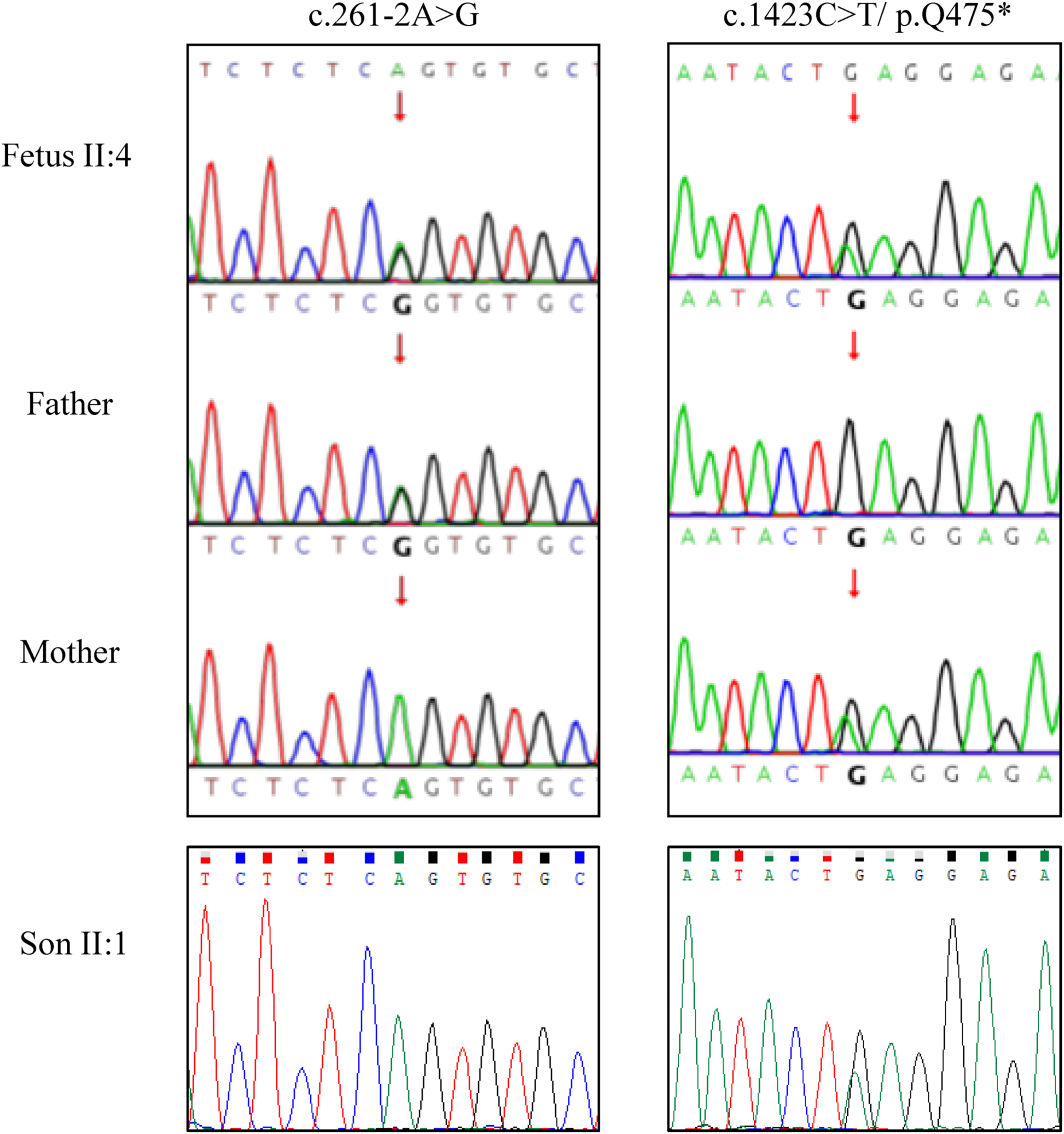
Sanger sequencing validated the compound heterozygous variants of *B3GALNT2* in the porband (the fetus II:4). The c.261‐2A>G variant is of paternal origin, and the c.1423/p.Q475* variant is of maternal origin.

Exome sequencing (ES) with Sanger sequencing validation confirmed the compound heterozygous variants of *B3GALNT2* (GenBank accession: NM_152490.5) in the fetus, namely the c.1423C > T (p.Gln475Ter) of maternal origin and the c.261‐2A > G of paternal origin. The heterozygous c.1423C > T variant was detected in the healthy son (II:1) (Figure 4). The c.1423C > T (p.Gln475Ter) variant has been reported previously, and classified as pathogenic in ClinVar database (https://www.ncbi.nlm.nih.gov/clinvar, Variation ID: 41939). The c.261‐2A > G variant destroys the canonical splice acceptor site in intron 2 and is predicted to cause abnormal gene splicing, producing incorrect mRNA subjected to nonsense‐mediated decay or carrying wrong protein‐encoding information.

## 4 CONCLUSION AND RESULTS

Our case study implies that *B3GALNT2* might be a causative gene for an encephalocele, providing new insights into the clinical spectrum of *B3GALNT2* mutations.

## 5 DISCUSSION

The c.261‐2A > G variant has an extremely low frequency^4^, ^5^, ^10^ in the public databases including the Genome Aggregation Database (gnomAD), the Exome Aggregation Consortium (ExAC) and the 1000 Genomes (1000G). In a recent study, the c.261‐2A variant was detected in trans with c.1307A > G (p.Tyr436Cys) in a patient with MEB.^9^ According to the ACMG (American College of Medical Genetics and Genomics) guidelines, the c.261‐2A > G variant is regarded as pathogenic (evidence levels PVS + PM2 + PM3).^10^

Encephaloceles are uncommon congenital malformations of the neural tube, and the etiologies are not well understood. It is largely believed that encephaloceles are polygenic diseases.^11^, ^12^

The *B3GALNT2* gene is among the reported 18 genes that have been associated with dystroglycanopathies.^9^ Based on the origin of the genetic defect, dystroglycanophathies are generally classified as^1^ primary dystroglycanopathies associated with the human *DAG1* (dystrophin‐associated glycoprotein 1) gene, which encodes the precursor protein that is post‐translationally glycosylated and cleaved into α‐DG and β‐DG^2^; secondary dystroglycanopathies resulting from mutations in the glycosyltransferase genes, such as POMT1, POMT2, POMGnT1, POMGNT2, FKTN, FKRP, POMK, TMEM5, B3GALNT2, B4GAT1, and LARGE1^3^; tertiary dystroglycanopathies related to the genes involved in biosynthesis of donor substrates used by the glycosylation enzymes, including *ISPD*, *GMPPB*, *DPM1*, *DPM2*, *DPM3*, and *DOLK*. In addition to genetic heterogeneity, dystroglycanophathy also shows a wide clinical spectrum, which is summarized into the severe phenotype characterized by congenital muscular dystrophy (CMD) with brain and (or) eye involvement (e.g. Walker‐Warburg syndrome (WWS)), MEB or Fukuyama CMD (FCMD), the intermediate phenotype manifesting CMD with or without mental retardation, and the milder type, limb‐girdle muscular dystrophy (LGMD).^9^, ^13^

Previous studies have shown that individuals reported with *B3GALNT2* variants most commonly presented with the severe WWS or MEB/FCMD‐like phenotype, though with exceptions for milder/non‐syndromic phenotypes.^7^, ^8^, ^14^ The phenotype severity of B3GALNT2 variants can only be partially explained by the pattern of mutations. Biallelic lossof‐function (LOF) variants are most commonly seen in association with a WWS phenotype. Compound heterozygous missense variants are more often associated with a MEB/FCMD‐like disease. Remarkably, the combination of a missense variant and a frameshift variant leads to a range of variable phenotypes.^7^, ^8^

Though many patients with causative *B3GALNT2* mutations have been documented, prenatal cases are rarely reported. We have identified the compound heterozygous variants *B3GALNT2* NM_152490.5:c.[1423C > T (p.Gln475Ter)]; [261‐2A > G] in a fetus with encephalocele and hydrocephalus. In an earlier study, Stevens et al. first identified a patient with homozygous *B3GALNT2* c.1423C > T variant in a cohort of dystroglycanopathy with structural brain involvement.^7^ In a more recent study, Song et al. identified three patients with compound heterozygous *B3GALNT2* variants from a cohort of 143 patients with dystroglycanopathy. The three patients had a MEB phenotype, one of whom (a 1 year and 10 months old male) carried the compound heterozygous variants c.261‐2A > G and c.1307A > G (p.Tyr436Cys).^9^ Though brain involvement has been associated with the c.1423C > T (p.Gln475Ter) variant and the c.261‐2A > G variant in separate studies, our case first shows that compound heterozygosity of these two variants may cause an encephalocele (neural tube defect) and brain ventricle enlargement (hydrocephalus) in fetus. Recently, Wang et al. have linked *B3GALNT2* compound heterozygous variants c.[1A > G (p.Met1Val)]; [1151 + 1G > A] and c.[1052 T > A (p.L351Ter)]; [1068dupT (p.D357Ter)] with fetal brain structural abnormalities, such as enlarged bilateral ventricles and hypoplasia of midline structure.^15^ Our findings are in line the Wang's study, suggesting that *B3GALNT2* mutations, especially the loss‐of‐function mutations could be an etiology of congenital brain malformations. Further studies are warranted to reveal the pathological role of *B3GALNT2* in neural tube defects.

In summary, our study highlights *B3GALNT2* as a potential causative gene for an encephalocele (neural tube defect). This finding may contribute to prenatal screening/diagnosis and genetic counseling of fetal central nervous system malformations. The present case also emphasizes the clinical significance of genetic testing in childbearing‐age couples for the next pregnancy given the previous adverse pregnancy outcomes.

## AUTHOR CONTRIBUTIONS

**Dandan Ling:** Conceptualization; data curation; writing – review and editing. **Wan‐Qin Xie:** Conceptualization; writing – review and editing. **Xiao Mao:** Formal analysis; funding acquisition; methodology. **Shengzhi Yang:** Methodology; resources. **Haiyan Pang:** Methodology; validation; visualization. **Ping Yang:** Supervision; validation. **Ping Shen:** Project administration. **Yabing Tang:** Funding acquisition; project administration; supervision.

## FUNDING INFORMATION

This work was supported by the Major Scientific and Technological Projects for Collaborative Prevention and Control of Birth Defects in Hunan Province (2019SK1012). This work was also supported by the Clinical Research Center For Placental Medicine In Hunan Province (2021SK4020).

## CONFLICT OF INTEREST STATEMENT

The authors declare that they have no competing interests.

## CONSENT

Written informed consent was obtained from the patient to publish this report in accordance with the journal's patient consent policy.

## Data Availability

Data openly available in a public repository‐ The authors confirm that the data supporting the findings of this study are available within the article.
